# Boron Compounds Mitigate 2,3,7,8-Tetrachlorodibenzo-p-dioxin-Induced Toxicity in Human Peripheral Blood Mononuclear Cells

**DOI:** 10.3390/toxics12020098

**Published:** 2024-01-23

**Authors:** Mehmet Enes Arslan, Cem Baba, Ozlem Ozdemir Tozlu

**Affiliations:** Department of Molecular Biology and Genetics, Faculty of Sciences, Erzurum Technical University, 25050 Erzurum, Turkey; cem.baba39@erzurum.edu.tr (C.B.); ozlem.ozdemir@erzurum.edu.tr (O.O.T.)

**Keywords:** boron compounds, TCDD, genotoxicity, cytotoxicity, oxidative status

## Abstract

2,3,7,8-Tetrachlorodibenzo-p-dioxin (TCDD) stands as one of the most potent halogenated polycyclic hydrocarbons, known to inflict substantial cytotoxic effects on both animal and human tissues. Its widespread presence and recalcitrance make it an environmental and health concern. Efforts are being intensively channeled to uncover strategies that could mitigate the adverse health outcomes associated with TCDD exposure. In the realm of counteractive agents, boron compounds are emerging as potential candidates. These compounds, which have found applications in a spectrum of industries ranging from agriculture to pharmaceutical and cosmetic manufacturing, are known to modulate several cellular processes and enzymatic pathways. However, the dose–response relationships and protective potentials of commercially prevalent boron compounds, such as boric acid (BA), ulexite (UX), and borax (BX), have not been comprehensively studied. In our detailed investigation, when peripheral blood mononuclear cells (PBMCs) were subjected to TCDD exposure, they manifested significant cellular disruptions. This was evidenced by compromised membrane integrity, a marked reduction in antioxidant defense mechanisms, and a surge in the malondialdehyde (MDA) levels, a recognized marker for oxidative stress. On the genomic front, increased 8-OH-dG levels and chromosomal aberration (CA) frequency suggested that TCDD had the potential to cause DNA damage. Notably, our experiments have revealed that boron compounds could act as protective agents against these disruptions. They exhibited a pronounced ability to diminish the cytotoxic, genotoxic, and oxidative stress outcomes instigated by TCDD. Thus, our findings shed light on the promising role of boron compounds. In specific dosages, they may not only counteract the detrimental effects of TCDD but also serve as potential chemopreventive agents, safeguarding the cellular and genomic integrity of PBMCs.

## 1. Introduction

Dioxins represent a class of highly toxic and persistent organic pollutants pervasive in our environment, with a propensity to accumulate in the fatty tissues of animals. Dioxins primarily enter the human body via the food chain, and they can also be transmitted from a mother to her infant, predominantly through the placenta during pregnancy and through breast milk during breastfeeding [1]. Foremost among these is 2,3,7,8-tetrachlorodibenzo-p-dioxin (TCDD), acknowledged as the most toxic member of the dioxin family. TCDD is a well-known substance for its detrimental effects on the immune system, its ability to cause cancer, disrupt the endocrine system, induce cardiovascular toxicity, and its potential to trigger abnormalities in reproduction and development [2,3,4,5,6,7]. Experiments on laboratory animals have unveiled various physiological responses to TCDD, including immunotoxicity, neurological and behavioral abnormalities, the potential to induce cancer, and significant alterations in biochemical indicators [8]. In terms of mechanisms, the toxicity of TCDD is thought to primarily stem from the generation of reactive oxygen species (ROS) and the disruption of antioxidant defenses, resulting in extensive damage to cells and tissues [9,10]. Furthermore, the aryl hydrocarbon receptor (AhR) pathway, with its subsequent activation of CYP1A1 and 1B1 enzymes, plays a pivotal role in TCDD’s genotoxic effects, contributing to genomic instability and the development of tumors [11,12,13,14,15]. The widespread presence of dioxin in the environment serves as a clear indication of the ongoing, constant exposure experienced by humans. This emphasizes the urgent requirement for actions to reduce the potential harm it poses. In the pursuit of protective strategies, antioxidant compounds have recently gained significant attention. Their inherent capacity to counteract free radicals makes them promising candidates for mitigating the adverse effects caused by various harmful substances.

Boron (B) is an exceptional trace element that plays a crucial role in numerous biological functions across a variety of organisms [16]. Boron is not typically found in its elemental form in nature; instead, it is primarily obtained from dissolved boric acid in water and boron compounds derived from plants [17,18]. Two economically significant boron compounds, borax (BX) and ulexite (UX), are frequently found alongside other minerals, such as magnesium, sodium, and calcium [19,20,21]. Boron plays a vital role in cell division and growth in various higher animals, and deficiencies in this element can have adverse effects on the immune system, cognitive functions, skeletal health, and lipid metabolism [22,23,24,25]. Furthermore, boron demonstrates antioxidative properties, either by augmenting glutathione levels or facilitating the production of other ROS (reactive oxygen species) neutralizing agents [26]. Notably, boron deficiencies have been linked to elevated enzymatic activities of peroxidase, catalase, and superoxide dismutase (SOD) in specific biological contexts [27]. Experiments have shown variable effects of boron on enzymatic activities depending on the dosage, with enhancement at low doses and reduction at higher concentrations [28]. There is also compelling evidence of boron’s protective role against hepatic damage in animal models [29,30,31]. Contemporary research has broadened the scope, probing into the anticancer potential, antimicrobial properties, drug delivery capabilities, and biomolecular interactions of novel boron-based compounds [32,33,34,35,36].

Throughout recent years, the scientific community has witnessed a surge in research aimed at combating the deleterious effects of TCDD-mediated cytotoxicity. Numerous treatments have been probed, each exhibiting varying degrees of efficacy [37]. Regrettably, despite these exhaustive efforts, current therapeutic strategies remain somewhat deficient in fully curbing the toxicity elicited by TCDD [38]. One avenue that holds substantial promise is the exploitation of natural antioxidant molecules, which, when incorporated into our diet, can bolster the body’s defenses against such toxic challenges. As TCDD’s harmful effects are largely mediated through lipid oxidation and reactive oxygen species (ROS) production, neutralizing these pathways emerges as a logical counterstrategy [39]. Consequently, an intervention that adeptly mitigates both these mechanisms could prove invaluable in managing TCDD toxicity. Guided by this rationale, the present investigation sets out on an exploratory journey, aiming to ascertain the cyto- and genoprotective properties of BA, BX, and UX. Specifically, we are driven by the hypothesis that these compounds might offer a formidable shield against the cytotoxic, oxidative, and genotoxic onslaught instigated by TCDD exposure. 

## 2. Materials and Methods

### 2.1. Chemicals

As test substances, BX (Na_2_B_4_O_7_·10H_2_O, CAS No. 1330-43-4, 381.37 g·mol^−1^, ≥99.5% pure) and BA (H_3_BO_3_, CAS No. 10043-35-3, 61.83 g·mol^−1^, ≥99.9% pure) were purchased from Sigma-Aldrich (St. Louis, MO, USA), and UX (Na_2_O·2CaO·5B_2_O_3_·16H_2_O, CAS No. 1319-33-1, 405.21 g·mol^−1^, ≥98% pure) was supplied from Eti Mine Works in Turkey. 2,3,7,8-Tetrachlorodibenzo-p-dioxin; 2,3,7,8-TCDD was obtained from Sigma-Aldrich (St. Louis, MO, USA). All additional chemicals and reagents were of the analytical reagent grade and were obtained from commercial sources.

BA, BX, and UX were initially dissolved in distilled water, then diluted with medium to the desired final concentration. 

### 2.2. Blood Collection

Whole blood was obtained from 9 male volunteers (between the ages of 25 and 35 who had no prior history of exposure to any genotoxic agents) under sterile conditions into heparinized tubes by a specialist laboratory technician. Exclusion criteria were alcohol use, as well as medications use including supplements, herbal medications, and products of tobacco. All participants completed informed consent forms that were authorized by the Bioethics Committee of the Faculty of Health at Ataturk University. Then, each donor was venipunctured to obtain 5–20 mL of blood. Heparin was used as an anticoagulant for collecting the blood samples in tubes.

### 2.3. PBMC Isolation

Under sterile circumstances, PBMCs were collected by density gradient centrifugation of heparinized venous blood over Ficoll-Paque (Sigma, St. Louis, MO, USA). The obtained cells were rinsed three times in PBS and then suspended in RPMI-1640 (Gibco, Waltham, MA, USA) with 1% penicillin/streptomycin (Sigma) and 10% fetal bovine serum (FBS, Gibco, Waltham, MA, USA). The hemocytometer was used to count the PBMC, and 5 × 10^6^/mL of the total number was used [40].

### 2.4. Determination of TCDD Toxicity

The toxicity of TCDD was determined in PBMCs using an MTT assay. Briefly, PBMCs were grown in a 96-well microplate containing growth media and various doses of TCDD (0–2 mg/L) for 48 h. Following the incubation period, a solution of MTT (5 mg/mL MTT in PBS) was applied to each well at a 1:10 ratio. After 3 h, the microplates were subjected to centrifugation at 800 g for 5 min, and then 150 μL of dimethyl sulfoxide (DMSO) was added to dissolve any formazan crystals. The absorbance at a wavelength of 570 nm was measured using a microplate spectrophotometer (Synergy-HT; BioTek, Winooski, VT, USA). The results are expressed as a percentage of viable cells, and values represent an average of 4–6 replicates. Untreated cells served as the negative control (NC). 

### 2.5. Cell Viability Assay

PBMCs were grown in a 96-well microplate containing growth media. The cells were exposed to TCDD and, 6 h later, were treated with various doses of B compounds (2.5, 5, and 10 mg/L) for 48 h. The selection of B compound concentrations was based on our prior research findings [41,42]. MTT solution (5 mg/mL MTT in PBS) was applied to each well at a ratio of 1:10 at the end of incubation. After 3 h, the microplates were centrifuged at 800× *g* for 5 min, and 150 μL DMSO was added to dissolve any formazan crystals. A microplate spectrophotometer (Synergy-HT; BioTek, Winooski, VT, USA) was used to measure the absorbance at a wavelength of 570 nm. The results are presented as a percentage of viable cells. Values were determined as the average of 4–6 repeats. Untreated cells were used as a negative control (NC) and cells treated with 1% Triton X-100 were used as a positive control (PC) [40]. 

### 2.6. Membrane Integrity Assay

Membrane integrity was assessed using the Cytoselect^TM^ MTT Cell Proliferation Assay (Cell Biolabs, Inc., San Diego, CA, USA) kit in accordance with the manufacturer’s recommendations. The growth media and various doses of B compounds (2.5, 5, and 10 mg/L) both alone and against TCDD-induced toxicity were added to a 96-well microplate to cultivate PBMCs for 48 h. At the end of incubation, 90 mL of the supernatant and 10 mL of the test reagent were combined and incubated at 37 °C for 30 min in a fresh microplate. The optical density was determined using a microplate spectrophotometer (Synergy-HT; BioTek, Winooski, VT, USA) at a wavelength of 450 nm. The results were presented as an LDH activity %. Values are the average of 4–6 replicates. Untreated cells were used as negative control (NC) and cells treated with 1% Triton X-100 was used as a positive control (PC) [40,43].

### 2.7. Total Antioxidant Capacity Assay

PBMCs were cultured in a 96-well microplate containing the growth medium and various doses of B compounds (2.5, 5, and 10 mg/L) both alone and against TCDD-induced toxicity for 48 h. After incubation, the antioxidative potential of B compounds in plasma samples was assessed using the total antioxidant capacity (TAC) assay kit from Rel test Diagnostics^®^, Turkey, in accordance with the manufacturer’s instructions [42]. 

### 2.8. Membrane Lipid Peroxidation Assay

Malondialdehyde (MDA), a product of the thiobarbitiric acid (TBA) reaction, was used to measure lipid peroxidation [44]. Basically, PBMCs were cultured with various doses of B compounds (2.5, 5, and 10 mg/L) both alone and against TCDD-induced toxicity for 48 h. Then, 10% trichloroacetic acid (TCA) was added to 500 μL of cell lysate and subsequently heated to 90 °C for 15 min. Additionally, 1 mL of supernatant was combined with 1 mL of TBA after a ten-minute centrifugation, and the mixture was once more incubated at 90 °C for 15 min. In comparison to a blank, the absorbance of the MDA-TBA product at 532 nm was measured. The 1.56 × 10^5^ mol L^−1^ cm^−1^ MDA extinction coefficient was used to determine the amount of lipid peroxidation in cells as mM MDA per g. Hydrogen peroxide (H_2_O_2_) (25 μM) was used as a positive control.

### 2.9. Chromosomal Aberration (CA) Assay

Whole heparinized bloods were incubated in a complete media containing RPMI 1640 medium with 15% fetal bovine serum, l-glutamine, penicillin-streptomycin (1%), and phytohaemagglutinin (3%) at 37 °C for 72 h. After the beginning of culture, at 24 h, the cells were treated with different B compounds (2.5, 5, and 10 mg/L) both alone and against TCDD-induced toxicity (48 h treatment period). In addition, parallel tests were also conducted using a negative (untreated) and positive (treated with 0.2 μg/mL Mitomycin C (MMC)) control. Two hours prior to harvesting (i.e., the last 2 h of the 72 h incubation period), Colchicine (0.02 g/mL) was given to the culture in order to stop the dividing cells at metaphase. Following the 72 h of incubation time, the cells were fixed in a methanol–acetic acid solution and treated with a hypotonic solution (0.075 M KCl). The cells were then centrifuged and placed on clean glass slides. To stain the slides, Giemsa in phosphate buffer (pH 6.8) was used. The study analyzed specific types of chromosomal aberrations, including breaks, gaps, fragments, dicentrics, rings, translocations, and inversions, as per the guidelines outlined in the Environmental Health Criteria 46 for environmental monitoring of human populations [40]. 

### 2.10. 8-Hydroxy-2′-deoxyguanosine Assay

To measure the levels of 8-hydroxy-2′-deoxyguanosine in the cultures, DNA/RNA Oxidative Damage (High Sensitivity) ELISA Kits were obtained from Cayman Chemical (Ann Arbor, MI, USA). Much research is being performed on the use of this approach since it is a competitive assay that can be used to quantify 8-OH-dG in homogenates and identify both free 8-OH-dG and DNA-incorporated 8-OH-dG. The entire process was completed in line with the provider manual. Briefly, DNA was isolated from the cell cultures by using the Genomic DNA Purification Kit (Thermo Fischer Scientific^®^, Waltham, MA, USA). Then, 50 μL of the ELISA buffer was mixed with 50 μL of the sample or 50 μL of the standard in the plate. Then, 50 μL of the monoclonal antibody was added into the wells. After the incubation period, plate contents were removed and rinsed with wash buffer. Then, 200 μL of Elmann’s Reagent was added to each sample and incubated for 90 min using an orbital shaker. At the end of incubation, the plate was read at a wavelength of 450 nm using a microplate reader [42]. 

### 2.11. Biochemical Analyses

The assessment of various biochemical markers is crucial in understanding cellular and molecular processes. Among these, the cytochrome C reduction rate plays a pivotal role. This rate was meticulously determined after its inhibition by the superoxide radical. Such a determination required measurements at a specific wavelength of 550 nm [45]. In parallel, the activity of catalase (CAT), an essential enzyme responsible for breaking down hydrogen peroxide, was evaluated. For this purpose, the enzyme extract was integrated with an H_2_O_2_-phosphate buffer. A spectrophotometer was then used to calculate the resulting activity at 240 nm, which was quantified and expressed in terms of µmol/min/mg protein [46]. Moving forward, the role of glutathione peroxidase (GPx) cannot be overlooked. Its activity was deciphered by monitoring the oxidation of NADPH. This oxidation process was observed at a wavelength of 340 nm and was notably affected by the presence of t-butyl [47]. Glutathione (GSH), a significant antioxidant in the body, had its levels measured at 412 nm. This measurement technique was derived from the method proposed by Beutler and Kelly in 1963, which hinges on the distinctive yellow coloration produced from the reaction of 5,5′-dithiobis-(2-nitrobenzoic acid) (Ellmann’s solution) with sulfhydryl groups [48]. Tumor necrosis factor alpha (TNF-α) is a critical cytokine involved in inflammatory processes. To accurately investigate its levels, an ELISA kit (YLA0053FI, YLbiant) was employed according the manufacturer’s instructions. Similarly, interleukin-6 (IL-6), another essential cytokine that plays a pivotal role in inflammation and immune response, was assessed using the IL-6 ELISA kit (YLA 0017FI, YLbiant).

### 2.12. Statistical Analysis

The data are presented as values of mean ± standard error mean (*SEM*), which were obtained from experiments carried out in triplicate. The differences in variance were analyzed statistically using a one-way analysis of variance (ANOVA) test by GraphPad prism 8.0 statistics software (GraphPad, La Jolla, CA, USA). Tukey’s test was used as a post hoc.

Dunnett’s test for multiple comparisons was performed for biochemical analysis. The differences were considered significant when *p*  <  0.05.

## 3. Results

### 3.1. Effects of B Compounds on TCDD-Mediated Cytotoxicity

**Figure 1:**
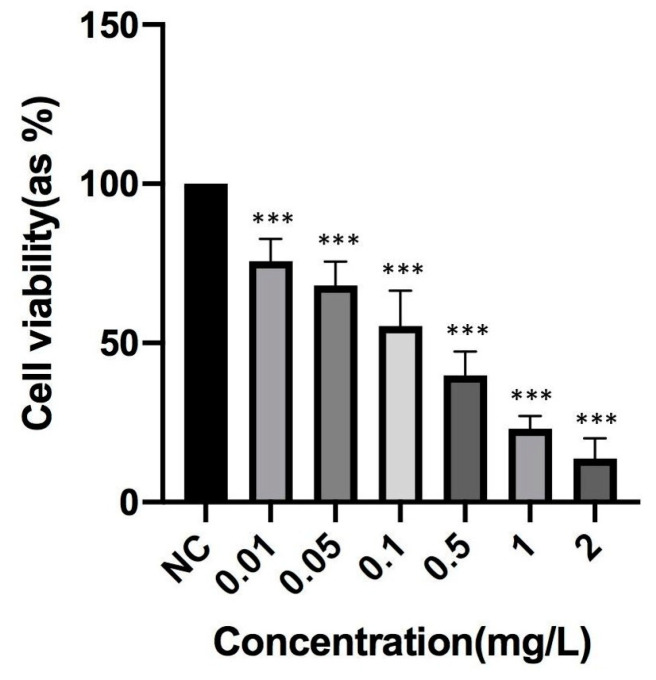
The effect of 48 h treatment with different concentrations of tetrachlorodibenzo-p-dioxin (TCDD) on peripheral blood mononuclear cell (PBMC) viability. Data are presented as mean ± SEM. Significant difference *p* < 0.001 (***) compared to the negative control group (NC: negative control, SEM: standard error of the mean).

**Figure 2:**
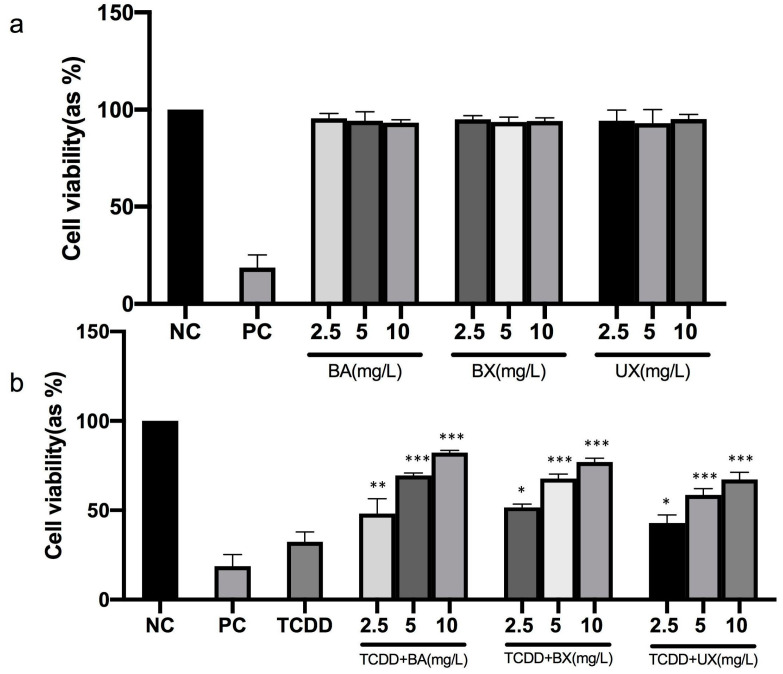
The effect of 48 h treatment with different concentrations of boric acid (BA), borax (BX), or ulexite (UX) (2.5–10 mg/L) (**a**) and 48 h co-treatment with different concentrations of BA, BX, or UX (2.5–10 mg/L) against tetrachlorodibenzo-p-dioxin (TCDD) (0.1 mg/L) toxicity (**b**), on peripheral blood mononuclear cell (PBMC) viability. Data are presented as mean ± SEM. Significant difference *p* < 0.05 (*), *p* < 0.01 (**), and *p* < 0.001 (***), compared to the TCDD-only group (NC: negative control, PC: positive control, SEM: standard error of the mean).

The TCDD cytotoxic concentration for PBMC was determined by MTT assay (Figure 1). A TCDD concentration of 0.1 mg/L, equivalent to an IC_50_ (inhibitory concentration for 50%) value, was chosen for subsequent investigations. PBMC cells were exposed to various concentrations of B-containing compounds to assess their safety. The findings indicated that the application of different B-containing compounds did not alter cell viability in PBMC cells after 48 h (Figure 2a).

This study investigated the potential protective effects of B compounds on PBMC cells in response to TCDD-induced toxicity. The results revealed that treatment with various B-containing compounds significantly reversed the TCDD-induced reduction in cell viability at 48 h of co-treatment, at concentrations of 2.5, 5, and 10 mg/L (*p*  <  0.05). When compared to BX and UX, BA exhibited a more pronounced effectiveness in mitigating TCDD toxicity (as shown in Figure 2b).

**Figure 3:**
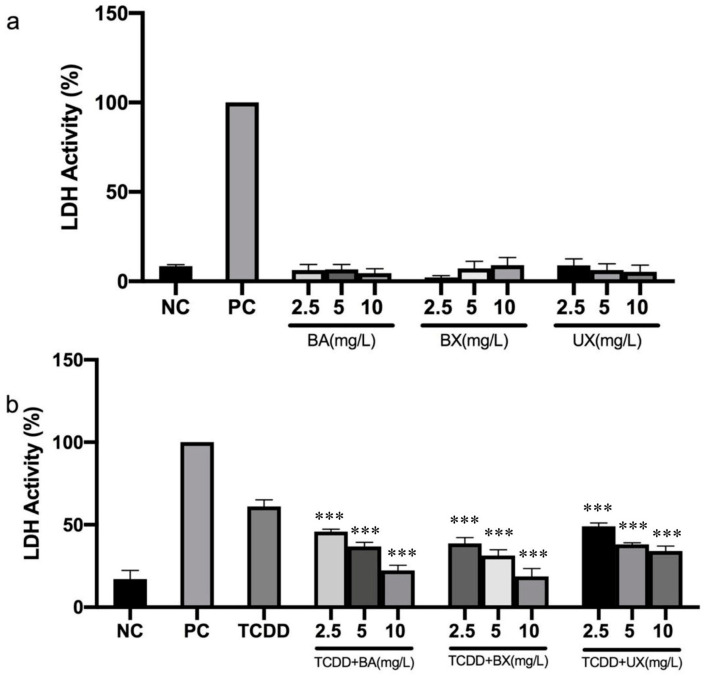
The activity of LDH released into the supernatant of peripheral blood mononuclear cell (PBMC) cultures 48 h after treatment with boric acid (BA), borax (BX), or ulexite (UX) (2.5–10 mg/L) (**a**) and 48 h co-treatment with different concentrations of BA, BX, or UX (2.5–10 mg/L) against tet-rachlorodibenzo-p-dioxin (TCDD) (0.1 mg/L) toxicity (**b**). Data are presented as mean ± SEM. Significant difference *p* < 0.001 (***), compared to the TCDD-only group (NC: negative control, PC: positive control, SEM: standard error of the mean).

The assessment of membrane integrity, as indicated by LDH activity, showed that the activity remained unchanged in cells treated with different B compounds. However, exposure to TCDD significantly elevated LDH activity, reaching 61.03% (Figure 3a). Treatment with various B compounds exhibited a potent protective effect against TCDD-induced LDH activity, reducing the values to 17.20% in the intervention group when compared to the TCDD-only group (*p* < 0.05). The results suggest that, depending on the dose, UX provides a better protection of membrane integrity against TCDD-induced toxicity compared to BA and BX (as illustrated in Figure 3b).

### 3.2. Effects of B Compounds on TCDD-Mediated Oxidative Stress

As indicated in Table 1, exposure to TCDD significantly elevated the levels of MDA and reduced the level of TAC. Furthermore, the application of B compounds did not lead to significant changes in the levels of MDA and TAC when compared to the control group. However, co-treatment with B compounds and TCDD notably decreased the MDA level and increased the TAC level (Table 1; *p* < 0.05). In comparison to other compounds, BX demonstrated greater effectiveness than BA and UX in enhancing the activities of antioxidant enzymes and reducing MDA levels in response to TCDD-induced toxicity.

### 3.3. Effects of B Compounds on TCDD-Mediated Genotoxicity

**Figure 4:**
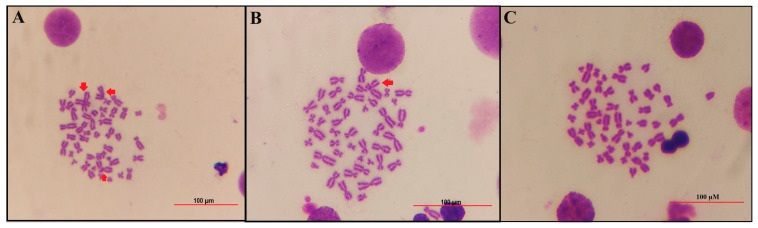
Sample images of metaphase plates investigated by using the chromosomal aberration test. Image of CA test performed on PBMC cultures treated with (**A**) TCDD (0.1 mg/L) only, (**B**) TCDD (0.1 mg/L) + boric acid (10 mg/L), (**C**) negative control (no treatment). Red arrows show translocations and breaks in (**A**)**,** and translocation in (**B**).

To assess whether B compounds could prevent genotoxicity induced by TCDD, chromosomal aberration (CA) and 8-OH-dG assays were conducted. Table 2 and Figure 4 presents the CA observed in TCDD-treated PBMCs. Our results demonstrated that TCDD (0.1 mg/L) significantly increased the frequency of CA and the 8-OH-dG level, confirming DNA damage. In contrast, the treatment of PBMCs with the pure B compounds did not exhibit any genotoxic effects when compared to the control group. Additionally, our findings indicated that B compounds have a mitigating effect on TCDD-induced genotoxicity. Depending on the dosage, BX offers greater protection against TCDD-induced genotoxicity compared to BA and UX. 

**Figure 5:**
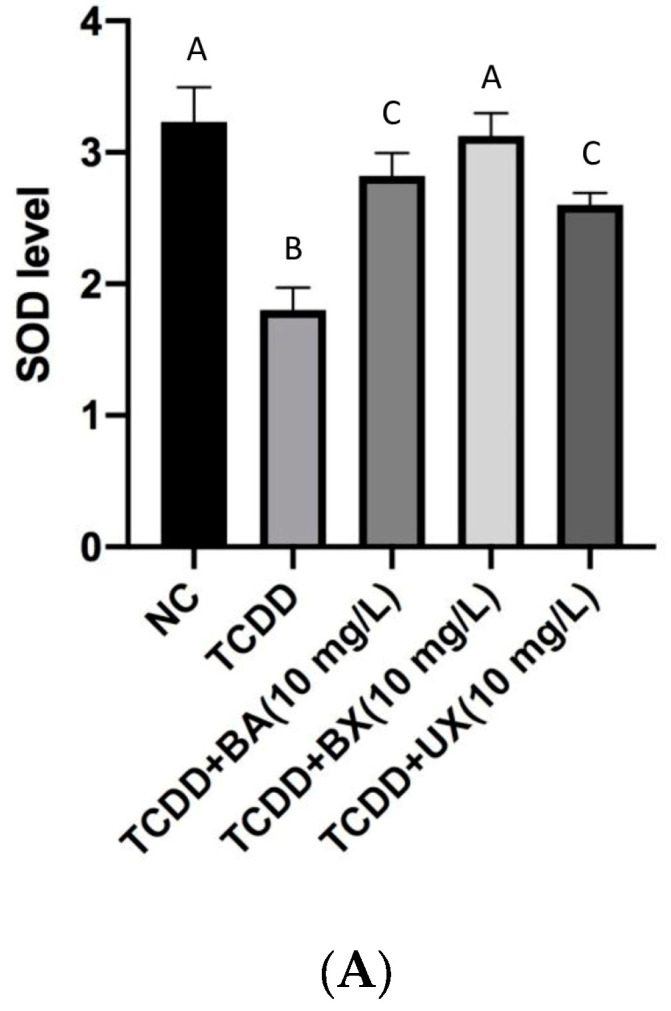

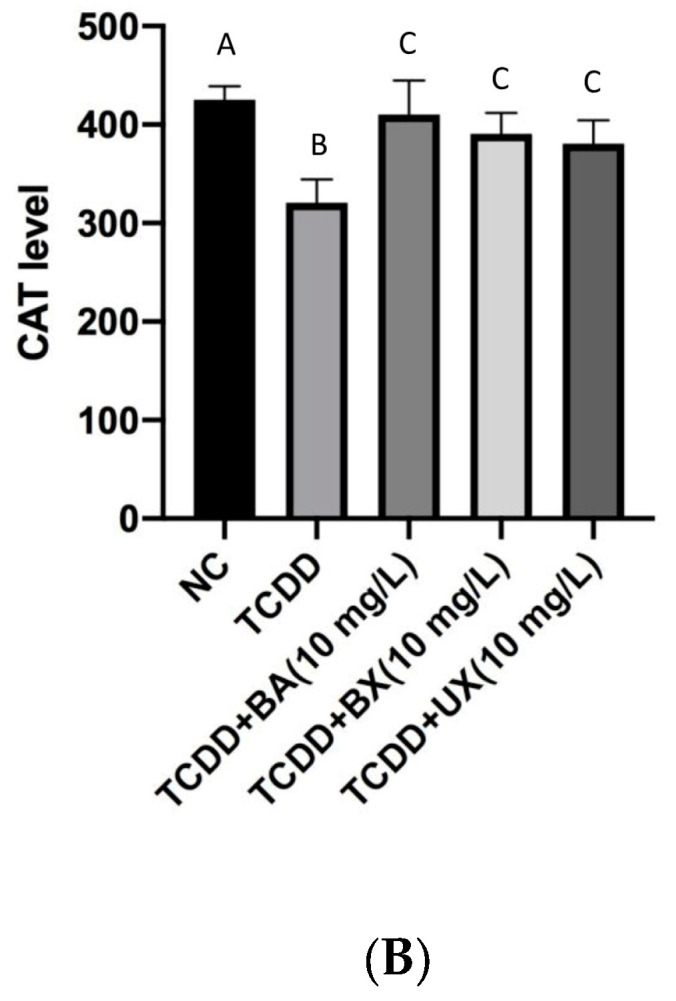

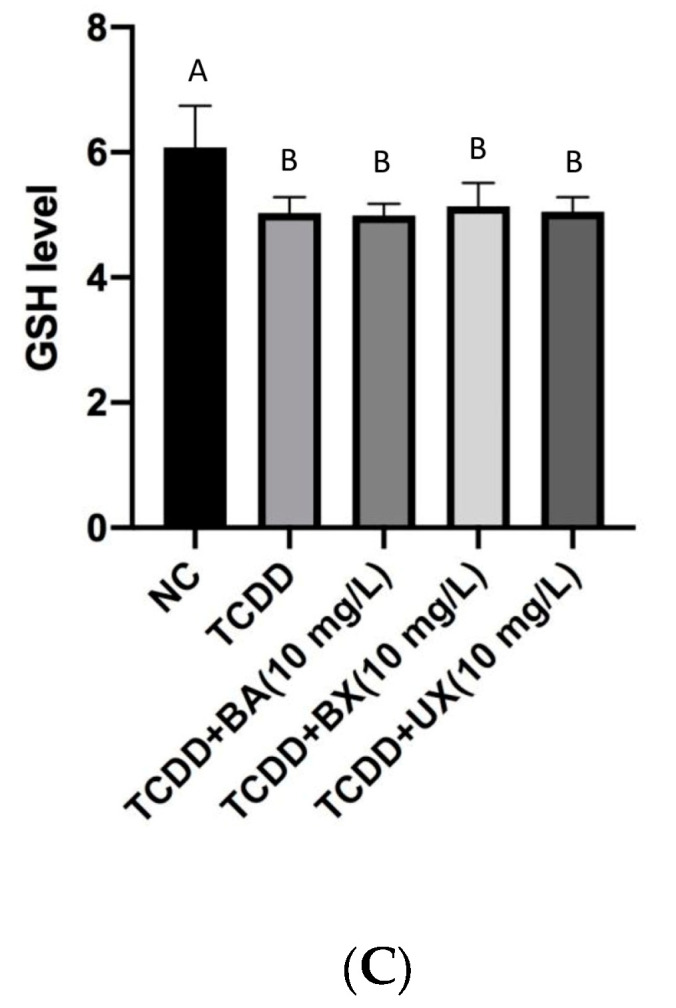

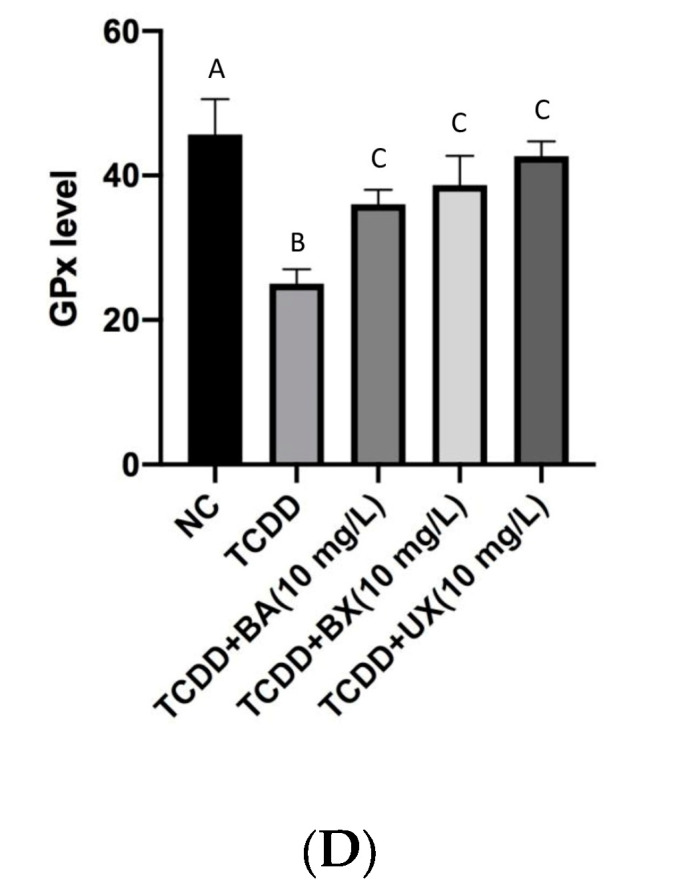
Effect of exposure to TCDD (0.1 mg/L) with BA, BX, and UX (10 mg/L) for 24 h on (**A**)—SOD (U/g tissue), (**B**)—CAT (U/g tissue), (**C**)—GSH (mg/g tissue), and (**D**)—GPx (mg/g tissue) in PBMC cultures. One-way ANOVA and Dunnett tests were used for multiple comparison. Letters (A–C) show statistically significant results compared to each other.

The results of TCDD exposure, in conjunction with BA, BX, and UX, on the superoxide dismutase (SOD) activity in PBMC cultures were investigated over a duration of 24 h. The baseline SOD activity in the negative control (NC) group hovered around 3.5 U/g tissue. When solely exposed to TCDD at a concentration of 0.1 mg/L, there was a noticeable reduction in the SOD activity, drawing close to 2.5 U/g tissue. Intriguingly, combining TCDD with BA at 10 mg/L exhibited an elevation in SOD levels, with the activity just above 3 U/g tissue. Even more compelling was the co-treatment of TCDD with BX at 10 mg/L, which mirrored the SOD activity akin to the NC group, nearly attaining 3.5 U/g tissue. The amalgamation of TCDD and UX at 10 mg/L saw the SOD values slightly surpassing 3 U/g tissue (Figure 5A). Also, the baseline CAT activity in the negative control (NC) group stood prominently at approximately 450 units. A stark contrast was observed when the cultures were exposed purely to TCDD, which resulted in a significant decline in CAT activity, bringing the value close to 250 units. However, the scenario shifts when introducing other compounds in conjunction with TCDD. The combination of TCDD with BA at 10 mg/L indicated a revival in CAT levels, with the activity hovering around 400 units. A similar bolstering effect was observed with the co-treatment of TCDD and BX at 10 mg/L, which brought the CAT activity back in line with the control group, nearing the 450 units mark. Notably, this restorative effect persisted when PBMC cultures were subjected to a mix of TCDD and UX at 10 mg/L, with CAT values remaining consistent with the control, approximating 450 units (Figure 5B). Moreover, a basal GSH concentration level was observed on the control group (NC) as approximating 6.5 mg/g tissue. However, a shift was evident when cultures were exposed exclusively to TCDD, resulting in a GSH concentration close to 5 mg/g tissue. This trend seemingly stabilized even when other compounds were introduced alongside TCDD. The combination of TCDD with BA, BX, and UX at 10 mg/L demonstrated a GSH level mirroring the sole TCDD exposure, situated around 5 mg/g tissue. To encapsulate, the GSH concentrations remained relatively consistent across the treatments, suggesting that these compounds might not offer a substantial protective or detrimental effect concerning GSH levels in the presence of TCDD (Figure 5C). Finally, glutathione peroxidase (GPx) levels in the negative control (NC) group robustly stand at approximately 55 mg/g tissue. When exposed solely to TCDD, there was a marked decrease, with GPx levels plummeting to nearly 35 mg/g tissue. This dynamic undergoes a transformation when integrating other compounds with TCDD. The merger of TCDD and BA at 10 mg/L showcases a slight recuperation in GPx concentrations, reaching close to 45 mg/g tissue. An analogous trend is observed when TCDD is paired with BX at 10 mg/L, maintaining the GPx values near the 45 mg/g tissue mark. However, an intriguing shift arises with the combination of TCDD and UX at 10 mg/L. This pairing witnesses a GPx level approaching the 50 mg/g tissue mark, indicating a somewhat enhanced recuperative effect compared to the BA and BX combinations (Figure 5D). 

**Figure 6:**
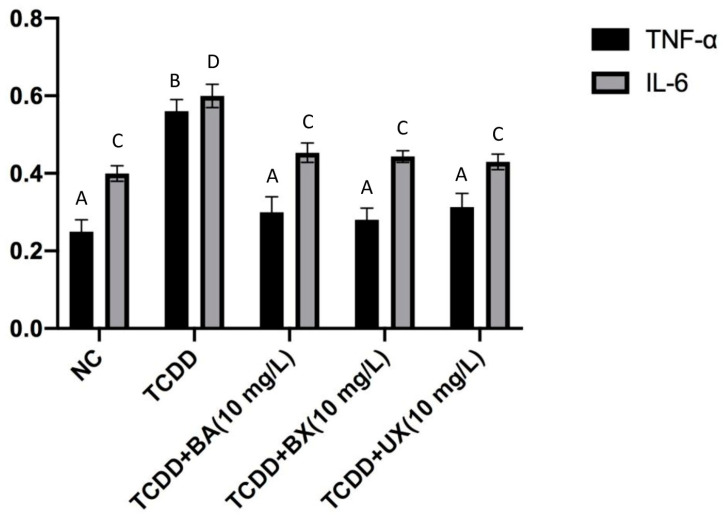
Effect of exposure to TCDD (0.1 mg/L) with BA, BX, and UX (10 mg/L) for 24 h on TNF-α and IL-6 levels in PBMC cultures. Letters (A–C) show statistically significant results compared to control. One-way ANOVA and Dunnett tests were used for multiple comparison. Letters (A–D) show statistically significant results compared to each other.

In Figure 6, the influence of TCDD exposure, in conjunction with BA, BX, and UX, on the levels of TNF-α and IL-6 was shown on PBMC cultures over a span of 24 h. Starting with the control group (NC), the levels of TNF-α were discernibly lower, approximately around 0.25 units, compared to IL-6, which stood slightly above 0.3 units. On introducing TCDD alone, both cytokines exhibited a perceptible elevation. TNF-α levels rose to just below 0.5 units, whereas IL-6 underwent a more modest increase, reaching nearly 0.35 units. As we venture into the combined exposures, intriguing patterns emerge. The amalgamation of TCDD and BA at 10 mg/L resulted in a distinctive response for both markers. While TNF-α levels hovered around the 0.45 units mark, IL-6 showcased a more pronounced leap, reaching an apex close to 0.65 units, the highest level observed in the presented data. The dynamic changes were seen when TCDD was paired with BX at 10 mg/L. Both TNF-α and IL-6 mirror each other, aligning around the 0.4 units mark. This parity in cytokine levels hints at a possible harmonized cellular response to this particular combination. Finally, the combination of TCDD and UX at 10 mg/L showcased TNF-α and IL-6 levels comparable to those observed with the BX pairing, with both cytokines stabilizing around the 0.4 units mark. In summation, the sole introduction of TCDD prompted an elevation in both TNF-α and IL-6 levels within the PBMC cultures. The integrative exposures, especially with BA, evoked varied responses, with the BA combination inciting the most substantial upsurge in IL-6 levels. On the other hand, the BX and UX pairings seem to harmonize the cytokine response, with both TNF-α and IL-6 converging at similar concentrations. 

## 4. Discussion

The primary objective of the present research was to assess the safeguarding impact of B compounds against dioxin-induced toxicity in PBMCs. In our investigation, the application of B compounds at concentrations ranging from 2.5 to 10 mg/L did not exhibit any inhibitory effects on PBMCs. Additionally, they significantly mitigated the cytotoxic effects triggered by TCDD at a concentration of 0.1 mg/L. This protective effect has been corroborated in previous studies using robust methodologies. In the research carried out by Atamanalp and colleagues, the simultaneous exposure of BX with acrylamide (AA) resulted in oxidative stress and the death of rainbow trout cells. Additionally, it lowered the baseline apoptosis of these cells by decreasing caspase-3 activity [49]. 

In a separate study, BX exhibited a beneficial impact on copper-induced kidney damage by promoting antioxidant actions and down-regulating gene expressions (specifically hsp70 and CYP1A), as indicated by reductions in MDA, caspase-3, and 8-OHdG levels [50]. The hepatoprotective characteristics of boric acid have also been documented in rat liver cells against aluminum toxicity [51]. 

TCDD exposure leads to tissue damage by elevating lipid oxidation and reducing the activity of antioxidant enzymes [52,53,54]. TCDD acts as a potent ligand for the aryl hydrocarbon receptor (AHR), with a strong affinity, causing an increase in the production of the CYP1A enzyme and disrupting the balance of oxidation and reduction. Additionally, TCDD hinders mitochondrial functions, resulting in the generation of reactive oxygen species (ROS) [55,56].

The harmful effects resulting from oxidative stress can be reduced through the use of antioxidant therapy. We conducted an investigation to determine whether various B compounds could mitigate the oxidative stress induced by TCDD in PBMCs, as shown in Table 1. We observed that TCDD administration significantly reduced the total antioxidant capacity (TAC) while substantially increasing the malondialdehyde (MDA) level. Consequently, oxidative damage exceeded the antioxidant defense mechanisms, resulting in tissue damage. In the co-treatment groups (BA  +  TCDD, BX  +  TCDD, UX  +  TCDD), MDA levels decreased, TAC levels increased significantly, and the damage caused by TCDD was noticeably alleviated by different B compounds. Moreover, B compounds had no impact on TAC and MDA levels in PBMCs. The rise in MDA levels is considered a crucial marker of oxidative stress. MDA is a byproduct of lipid peroxidation, and it serves as a fundamental indicator for evaluating the oxidative condition [57]. Likewise, a decrease in antioxidant levels leads to the occurrence of oxidative stress. Consequently, the activities of antioxidant enzymes play a significant role in cellular defense. Reports suggest that compounds containing boron have the potential to enhance the activity of antioxidant enzymes by reducing lipid peroxidation [58]. Another study indicates that boron treatment can effectively limit oxidative damage by increasing the body’s glutathione stores and inhibiting the formation of other reactive oxygen species [59]. These boron-containing compounds may enhance antioxidant enzyme activity through various pathways. Additionally, it has been reported that boron can induce structural changes in proteins, providing protection to organs against oxidative stress [60]. Furthermore, boron is believed to reduce intracellular levels of reactive oxygen species by boosting antioxidant enzyme activity [60]. In a separate study, the application of high doses of boron was found to reduce damage resulting from oxidative stress [61]. Our findings align with the existing literature; however, further research is imperative to uncover the precise molecular mechanisms involved.

The processes behind DNA damage brought on by TCDD have been well-documented. TCDD is not considered to be genotoxic; its potential to cause cancer is believed to arise from oxidative harm and the promotion of tumors through mechanisms that disrupt apoptosis [62,63,64]. These effects are likely connected to the activation of the aryl hydrocarbon receptor (AhR), as reported by the National Toxicology Program (NTP) in 2016 [65]. Nonetheless, the impact of B compounds on TCDD-triggered DNA damage is not fully understood. In order to explore this potential effect, we conducted a chromosomal aberration (CA) and 8-OH-dG assay. Furthermore, our results demonstrated a decrease in CA occurrence and 8-OH-dG levels induced by TCDD in PBMCs after exposure to B-containing compounds, affirming their genoprotective abilities, which align with findings from other studies [21,49,50]. B is regarded to be one of the substances that can reduce mutagen-induced mutagenicity [66,67,68]. Turkez et al. [66] proposed that boric acid can protect human lymphocytes from paclitaxel-generated genotoxicity. Turkez et al. [67] revealed in another investigation that B compounds (5–20 ppm) significantly mitigated the genotoxic effects of modest concentrations of heavy metals. The major harmful consequences of rising metal compound concentrations, according to their results, entail decreasing antioxidant enzyme levels. Furthermore, it was discovered that BX inhibits the rate of micronucleus (MN) and sister chromatid exchange (SCE) formations induced by AFB1 [68]. In V79 cell cultures, pretreatment of cells with modest concentrations of B (2.5 and 10 μM) revealed that B had a considerable protective effect against genotoxicity caused by cadmium chloride (CdCl_2_) and lead chloride (PbCl_2_) [68]. In our present study, we demonstrated that all concentrations of B compounds effectively counteracted the genotoxic effects of TCDD. The inhibitory effect increased in all co-treatment groups involving B compounds and TCDD. Based on these results, the observed advantages can be attributed to the antioxidant properties of B.

The superoxide dismutase (SOD) activity in PBMC cultures, following exposure to various compounds including TCDD, BA, BX, and UX, paints a complex picture of cellular oxidative stress dynamics. SOD, a pivotal antioxidant enzyme, plays an essential role in neutralizing the potentially damaging reactive oxygen species (ROS) generated within cells [69]. The observed decrease in SOD activity upon singular TCDD exposure aligns with the previous literature suggesting that TCDD might exert oxidative stress by hampering the cell’s antioxidant defence mechanism [70]. Interestingly, the modulating effect of BA, BX, and UX on SOD activity, in the presence of TCDD, provides avenues for deeper exploration. Some studies have reported that certain compounds can exhibit restorative effects on cellular antioxidant systems when challenged with toxic agents [71]. The near normalization of SOD levels, especially in the BX co-treatment scenario, warrants further investigations into the underlying molecular pathways. Catalase (CAT) is another vital antioxidant enzyme responsible for the detoxification of hydrogen peroxide within cells [72]. The diminished CAT activity post TCDD exposure resonates with the known detrimental effects of this compound. However, the revival of CAT levels upon co-treatment with BA, BX, and UX accentuates the protective potential of these compounds, possibly by counteracting the oxidative stress induced by TCDD. The relative consistency in glutathione (GSH) levels across all treatments, despite its pivotal role as a primary antioxidant in cellular systems [73], suggests that the impact of these compounds, specifically concerning GSH, might be minimal or compensated by other cellular mechanisms. Conversely, the intriguing dynamics surrounding glutathione peroxidase (GPx) levels open up new research prospects. GPx’s role in detoxifying peroxides and maintaining the cellular redox balance makes its modulation by TCDD and other compounds especially significant [74]. The enhancement in GPx levels with the TCDD and UX combination, in particular, indicates potential synergistic protective effects that could be of therapeutic interest. The present findings elucidate the modulatory influence of TCDD, a well-known environmental contaminant, on the production of key inflammatory cytokines, TNF-α and IL-6, in PBMC cultures. The amplification in cytokine expression observed upon TCDD exposure aligns with the literature indicating that TCDD can activate the aryl hydrocarbon receptor (AhR), leading to inflammatory responses [75]. This AhR activation triggers downstream signaling cascades that can result in the production of inflammatory cytokines [76]. The intriguing synergy observed upon combined exposure of TCDD with BA, which showed heightened IL-6 levels, might suggest an interplay between their respective signaling pathways. Environmental pollutants and their combined effects have garnered significant attention, especially since combined exposures can result in augmented effects compared to individual exposures [77]. The pronounced IL-6 response in the TCDD and BA combination underscores the importance of understanding combinatorial effects, particularly in environmental toxicology. Equally compelling is the harmonized cytokine response observed in the presence of BX and UX combined with TCDD. The stabilization of TNF-α and IL-6 levels in these settings might hint at a regulatory mechanism where BX and UX modulate the TCDD-induced responses. Such interactions have been previously reported for certain chemicals, where they influence the activation or suppression of specific signaling pathways [78].

In summary, this research has demonstrated that B compounds, up to a concentration of 10 mg/L, do not harm membrane integrity or lead to cell death in PBMCs. All tested concentrations enhance cellular antioxidant defenses and reduce MDA-induced oxidative stress. No genotoxic effects were observed with the application of B compounds. Moreover, B compounds were effective in reversing DNA damage caused by TCDD. However, there remain several unanswered questions regarding the toxicity of other B-containing compounds and the safe dosage range for humans. Nevertheless, the potential of B for its robust ameliorative and protective properties warrants in-depth investigation. This can be accomplished through translational studies utilizing animal models and comprehensive omics techniques. These endeavors will provide new insights into the effective and safe use of B for protection against environmental contaminants.

## 5. Conclusions

In drawing this research to a close, it becomes evident that B compounds serve as a notable shield against the detrimental effects of TCDD on PBMCs. Delving deeper into the findings, it is remarkable to see that B compounds, even at concentrations as high as 10 mg/L, remain benign, leaving the membrane integrity of PBMCs unscathed and preventing cellular demise. This not only underscores their safety at these levels but also emphasizes their potential therapeutic implications. Furthermore, our results throw light on the impressive role of B compounds in augmenting the cellular antioxidant defenses. Such an enhancement is crucial in combating the oxidative damage triggered by MDA. The significance of this defense mechanism cannot be overstated, especially when considering the broader cellular mechanisms at play and the profound implications of unchecked oxidative stress on cellular health. Another cornerstone of our study is the revelation that B compounds possess potent anti-genotoxic capabilities. TCDD’s genotoxic aftermath was considerably mitigated upon the application of B compounds. This leads to an optimistic perspective on B compounds’ potential applications, especially in environments rife with genotoxic agents. Equally reassuring is our observation that B compounds, in isolation, do not introduce any genotoxic perturbations, ensuring their safety profile in this regard. However, as with any scientific exploration, this study opens up more avenues for inquiry than it closes. For instance, questions about the overarching toxicity profiles of diverse B-containing compounds and the demarcation of safe dosage thresholds for human applications are still looming large. This underscores the need for continued research in this domain. Given B compounds’ compelling protective and ameliorative characteristics unearthed in this study, they beckon more intensive and exhaustive research. A plausible next step could be the deployment of translational research paradigms that leverage animal models, which could yield insights into the in vivo ramifications and potential therapeutic applications of B compounds. Moreover, harnessing the power of advanced omics techniques can potentially unveil the intricate cellular and molecular dialogues orchestrated by B compounds. In the larger scheme of things, these concerted research efforts can provide a more holistic understanding of B compounds and facilitate the formulation of robust guidelines. Such guidelines would be instrumental in harnessing the protective potential of B compounds, ensuring their safe and efficacious application in shielding humans from the ubiquitous environmental contaminants that mar our modern world.

## Figures and Tables

**Table 1:** TAC and MDA levels in cultures 48 h after treatment with BA, BX, or UX (2.5–10 mg/L) (a) and 48 h co-treatment with different concentrations of BA, BX, or UX (2.5–10 mg/L) against TCDD (0.1 mg/L) toxicity. Significant difference * *p*  <  0.05 compared to the TCDD-only group.

Groups	TAC Level (mmol/L)	MDA Level (Micromol/L)
Negative Control	5.1 ± 0.91	342.5 ± 5.32
Positive Control	18.8 ± 1.22	1075.8 ± 11.23
TCDD	1.7 ± 0.08	892.1 ± 7.86
BA 2.5	5.5 ± 0.54	325 ± 2.45
BA 5	6.2 ± 0.45	329 ± 5.21
BA 10	6.4 ± 0.68	290 ± 4.36
BX 2.5	5.8 ± 0.98	335 ± 4.78
BX 5	6.3 ± 0.39	298 ± 3.26
BX 10	6.7 ± 1.01	303 ± 6.32
UX 2.5	5.2 ± 0.43	331 ± 3.89
UX 5	5.5 ± 0.56	325 ± 5.01
UX 10	5.8 ± 0.32	319 ± 3.21
TCDD + BA 2.5	2.4 ± 0.03 *	741 ± 2.98
TCDD + BA 5	2.9 ± 0.08 *	697 ± 3.21 *
TCDD + BA 10	3.4 ± 0.27 *	592 ± 4.69 *
TCDD + BX 2.5	2.7 ± 0.02 *	782 ± 5.21
TCDD + BX 5	3 ± 0.09 *	575 ± 2.14 *
TCDD + BX 10	3. 2 ± 0.24 *	521 ± 2.67 *
TCDD + UX 2.5	2.3 ± 0.13 *	765 ± 4.21
TCDD + UX 5	2.7 ± 0.19 *	672 ± 1.98 *
TCDD + UX 10	2.9 ± 0.36 *	603 ± 3.17 *

Data are presented as mean ± SEM. * *p* < 0.05 as compared with the TCDD-only group, SEM: standard error of means, TAC: total antioxidant capacity, MDA: malondialdehyde.

**Table 2:** CA frequency and 8-OH-dG levels in cultures after 48 h treatment with BA, BX or UX (2.5–10 mg/L) (a) and 48 h co-treatment with different concentrations of BA, BX, or UX (2.5–10 mg/L) against TCDD (0.1 mg/L) toxicity.

Groups	CA/Cells	8-OH-dG Level Pmol/gDNA
Negative Control	2.40 ± 0.12	0.87 ± 0.02
Positive Controls	8.72 ± 0.45	4.35 ± 0.15
TCDD	7.15 ± 0.98	3.92 ± 0.09
BA 2.5	2.2 ± 0.04	0.78 ± 0.02
BA 5	2.1 ± 0.08	0.70 ± 0.04
BA 10	2.2 ± 0.23	0.83 ± 0.05
BX 2.5	1.9 ± 0.04	0.80 ± 0.05
BX 5	2.2 ± 0.67	0.68 ± 0.03
BX 10	1.8 ± 0.04	0.74 ± 0.09
UX 2.5	1.9 ± 0.07	0.72 ± 0.06
UX 5	2 ± 0.21	0.77 ± 0.02
UX 10	2.3 ± 0.34	0.71 ± 0.06
TCDD + BA 2.5	6.84 ± 0.78	3.11 ± 0.12 *
TCDD + BA 5	5.16 ± 0.67 *	2.62 ± 0.09 *
TCDD + BA 10	4.47 ± 0.89 *	2.28 ± 0.45 *
TCDD + BX 2.5	6.81 ± 0.27	3.40 ± 0.42 *
TCDD + BX 5	6.04 ± 0.34 *	2.52 ± 0.08 *
TCDD + BX 10	5.11 ± 0.09 *	2.09 ± 0.54 *
TCDD + UX 2.5	6.68 ± 0.17	3.44 ± 0.55 *
TCDD + UX 5	6.21 ± 0.48 *	3.08 ± 0.23 *
TCDD + UX 10	5.55 ± 0.42 *	2.77 ± 0.22 *

Data are presented as mean ± SEM. * *p* < 0.05 as compared with the TCDD-only group, SEM: standard error of means.

## Data Availability

The data that support the findings of this study are available from the corresponding author upon reasonable request.
